# Sanitary or Preventive Medicine

**Published:** 1898-10

**Authors:** Charles W. Heitzman

**Affiliations:** Chief of the Meat Inspection Service, New Orleans, La.


### SANITARY OR PREVENTIVE MEDICINE.

By Charles W. Heitzman, M.D.C.,
CHIEF OF THE MEAT INSPECTION SERVICE, NEW ORLEANS, LA.

In its primitive state, medicine comprised a recognition of the relative value of different articles of diet and a use of medicinal herbs and roots, and also of superstitious rites. Since the day of Jenner the trend of medicine has been toward prevention, and to-day modern medicine is prevention. At last has the time-honored axiom "an ounce of prevention is worth a pound of cure" become the cornerstone of the building. Slightly transpose, however, pounds of prevention, and you will not need an ounce of cure.

To win great battles you must have a well-trained army. The physician, no matter how well versed he may be in all rules of sanitation, must have the support of the public. That we may have an intelligent and efficient sanitation, it requires only a campaign of education on the part of the health authorities and the medical profession.

While, perhaps, the general public are aware that things which are not pleasant to the eye or nostrils are unsanitary, they should be taught that the hidden dangers are greatest. I refer to the food- and drink-supply. Let us consider, briefly, typhoid fever, the sanitary shame of American cities, a water-born disease—bad water-supply, bad sewerage, bad milk-supply; tuberculosis, the scourge of mankind, communicable from one animal to another by its products; trichinæ, and other parasitic diseases, and numerous nameless fevers, to which mankind is heir, can be directly traced to his food-supply. While a specific germ may not be isolated in each case, yet that silent, swift, and deadly ptomaïne is present, ever ready to produce anything from a simple bellyache to a fatal septic fever.

As a beginning, let us educate our fellow-man to the importance of having at least the three principal articles of his diet pure. First, meat. Teach him that a rigorous inspection of all meats intended for human consumption is absolutely necessary. Take him with you into the abattoirs; show him the numerous parasites with which the meat he is to eat is affected, and I warrant he will discontinue the use of liver for breakfast for several days, and when he begins to eat of it again he will be sure it has undergone a good inspection. Do not neglect to call his attention to tuberculosis, and that most of the cows having it come from dairies. This will make his milk a little unpalatable until he finds out that the dairies are as closely inspected as the meat. Show him some hog-cholera, cysticerci. Maybe some of his folks have had or have now a tapeworm; perhaps this will shed some light on its origin. Take him to the microscope, and let him see trichinæ; enlighten him as to their history, and, perhaps, without too great a stretch of his imagination, he can remember some peculiar cases of rheumatism that have occurred in his neighborhood. Do not forget the echinococcus. Some people read of more than they see; doubtless he has read of, if not seen, a case of pleurisy that proved a case of hydatid disease. Connect the two cases; it will bear fruit.

Do not neglect the cleanly part of the programme; call attention to clean floors, racks, and utensils, and to the large amount of pure, clean water used in slaughtering. Impress it on his mind, by seeing, that not only is disease looked after, but such meat as is allowed on the market does not get its final scrub with a dirty rag, instead of a nice clean brush being used; that the drainage of the abattoir is perfect, no filthy putrefying mass allowed under its floors to contaminate the meat, and that the wagons used for conveying the products of the slaughter to the market are likewise clean. Do not stop here; take him into the markets; prove to him that these are also under strict supervision; that no meat is allowed to be sold unless it has passed the ordeal witnessed by him.

That man's digestion will be better from this day on. He will eat more, sleep better, feel that at least one public office is doing all in its power to prolong his life; and, mind you, he will tell his neighbors. You will have some more visitors.

Next let us look at the bread-supply. A man should get sixteen ounces for a pound of the staff of life. The bakery and surroundings are of the utmost importance. Good drainage, good ventilation, and good water which should be analyzed from time to time. Cleanliness is here again a feature of great importance; everything should be immaculate. The operators should be bathed from time to time if they will not do so voluntarily. The flour used should be up to the standard. If the public pay for a first-class article of bread they should have it; not flour adulterated with cornmeal, clay, etc. Each loaf as it comes from the oven should be wrapped in specially-prepared paper, stamped with its proper weight and quality, and sealed. Take your friend in here; he will relish with avidity his roast-beef sandwich next day.

And now let us consider the last but not the least of the essentials of life. Out of the mouths of suckling babes I plead for pure milk. All babies, no matter of what nation, creed, or color, have our sympathy. Their helpless condition, if nothing more, appeals to us, and the wholesale slaughter of these helpless innocents by an impure food-supply is appalling. And were it so that not a single death be due to it, yet think of the number of sleepless nights of aches and pains that could be spared if we gave them a perfect food. And think! all these babies are destined some day, if we feed them properly, to become men and women, voters and mothers—a nation. The healthier the child the healthier the nation. The nearest, or as near, that nature has given us a perfect food, is fresh milk, and it remains with us as sanitarians to see that it is pure. Let us begin by providing comfortable quarters for the cows; good pure water, and plenty of it; cleanliness of surroundings, attendants, and utensils. Next go through the herd of cows, examine each one critically for disease. Do not limit yourself to tuberculosis. While examining the herd do not overlook the attendants. Milk is a great absorbent. And, lastly, examine the food; post yourself as to the best food for dairy-cows, and see that they get it. Do not stop at one examination; make them frequently. Submit the milk to frequent bacteriological and chemical analyses. Do the work honestly, thoroughly, and conscientiously, and the public will soon be with you.

Having once won their confidence and demonstrated to them the necessity for a strict sanitary supervision of the food-products, the victory is won. Other measures will follow; the death-rate will decrease, population increase, and such other radical changes ensue that could not be conceived by Verne in his wildest flights of imagination.

